# Experiences of women with disabilities accessing maternal and newborn health services in Mogadishu, Somalia

**DOI:** 10.1186/s12978-026-02361-6

**Published:** 2026-05-26

**Authors:** Muna Jama, Naoko Kozuki, Mamothena Carol Mothupi

**Affiliations:** 1 Somalia Program, International Rescue Committee, Airport Road, Mogadishu, Somalia; 2 International Rescue Committee, New York, USA; 3 International Rescue Committee, Nairobi, Kenya

**Keywords:** Maternal health services, Newborn health, Disability inclusion, Women with disabilities, Access to healthcare, Barriers to care, Somalia

## Abstract

**Background:**

Despite global commitments to inclusive and equitable maternal and newborn health (MNH) care, women with disabilities in fragile and conflict-affected settings remain overlooked. In Somalia, where maternal mortality is among the highest in the world, women with disabilities face systemic and structural barriers to accessing quality MNH services. This study examined experiences of and perspectives on equitable access to MNH care among women with disabilities in Mogadishu, Somalia.

**Methods:**

A qualitative descriptive study was conducted in Mogadishu between July and September 2024. We conducted in-depth interviews with women living with physical or visual impairments (*n* = 8) and with caregivers of women with hearing impairments or intellectual disabilities (*n* = 4). We also conducted key informant interviews with representatives of national disability organizations (*n* = 5). Interviews explored pregnancy and birth experiences, access to and quality of MNH services and recommendations for improvement. Data were analyzed using a primarily deductive thematic approach with inductive refinement, informed by Andersen’s Behavioral Model of Health Services Use and Morgan’s Gender Analysis Matrix to examine individual, facility and systemic-level determinants.

**Results:**

At the individual level, women with disabilities faced intersecting challenges related to gender, disability, poverty, unemployment, limited education, and social stigma. These factors pressured them into early marriage and childbirth to secure future caregiving support and social approval. Gendered power relations and unequal access to resources left many women relying on family members for transportation, financial support, and communication with healthcare providers, reducing their autonomy in MNH decision-making. Facility-level barriers included inaccessible infrastructure such as lack of ramps or adjustable beds, poor provider communication especially for patients with hearing impairments or intellectual disabilities, and provider stigma questioning women with disabilities’ right to motherhood. These experiences led many women with disabilities to avoid health facilities, relying instead on traditional birth attendants. Systemically, limited disability-inclusive MNH policy implementation, lack of data disaggregated by disability, and inadequate nationwide public awareness further compounded exclusion. Despite these constraints, women with disabilities identified free public health facilities and strong family and community support networks as key enabling factors.

**Conclusions:**

These findings demonstrate urgent MNH care gaps for women with disabilities in Mogadishu, Somalia and provide evidence for action. Priorities include disability-inclusive infrastructure, provider training, accessible communication approaches, stronger disability-disaggregated data systems, and women with disabilities participation in planning and service design.

## Abstract (plain language summary)

Women with disabilities have the same need for safe pregnancy, birth, and newborn care as other women, but disability-related challenges often make it harder for them to access health services. In Somalia, where many women already struggle to access maternal and newborn care, women with disabilities may face additional barriers, including unfair treatment, poverty, communication difficulties, and inaccessible health facilities. This study explored the experiences of women with disabilities in Mogadishu. Between July and September 2024, we spoke with women with physical or visual impairments, caregivers of women with hearing impairments or intellectual disabilities, and representatives from disability organizations. We asked about pregnancy and birth experiences, reaching services, treatment at facilities, and what could be improved. Women described obstacles such as poverty, limited education, unemployment, and stigma. Some felt pressure to marry and have children early to gain social acceptance and secure future support. Many depend on family for money, transport, and help communicating with health workers, reducing their independence in care decisions. At health facilities, women reported poor physical access, such as a lack of ramps and suitable beds, and communication that failed to meet their needs, especially for women with hearing impairments or intellectual disabilities. Some also faced disrespect and stigma, including being questioned about becoming mothers. These experiences sometimes led women to avoid facilities and rely on traditional birth attendants. Women also identified helpful factors, including free public health services and family and local support. They recommended accessible facilities, training in respectful maternity care provision, improved communication support, and the collection of disability-related data, local public awareness, and the involvement of women with disabilities in service planning.

## Background

Despite global declines in maternal and newborn morbidity and mortality, conflict-affected settings continue to report high rates [1]. For instance, the maternal mortality ratio in Somalia is estimated at 621 per 100,000 live births, compared with the global average of 197 per 100,000 live births [1]. Neonatal mortality also remains high at 35 per 1,000 live births [2]. These poor maternal and neonatal mortality indicators are exacerbated by prolonged conflict, recurring climate challenges, and chronic underfunding of the health system. The Somali health sector lacks sufficient emergency care for mothers and newborns and faces a shortage of skilled professionals, which results in inequitable access to health facilities [3]. Consequently, many women and newborns are unable to obtain essential health services [4]. Within this fragile health system context, Somalia’s 2024 National Disability Report estimates that 11.7% of adults aged 18 years and above live with disability, with higher prevalence observed in rural areas (13.5%) and among women (12.6%) [5]. This estimate should be interpreted with caution, as it pertains only to the adult population and is lower than the World Health Organization and World Bank global estimates of approximately 15% [6], suggesting possible underestimation in fragile and conflict-affected settings such as Somalia. Women with disabilities may face compounded barriers in accessing and utilizing quality maternal and newborn health (MNH) care in this setting [7]. Although women with disabilities have similar sexual and reproductive health needs as their peers, they often encounter additional barriers in accessing equitable and respectful MNH care. Systematic reviews and multi-country analyses conducted in sub-Saharan Africa have documented barriers affecting women with disabilities’ access to maternal and broader sexual and reproductive health services, including physical inaccessibility of facilities, communication barriers and limited provision of adapted information, and negative provider attitudes [8, 9]. These barriers can undermine privacy, informed decision-making, and respectful care, particularly when women must rely on companions to communicate or navigate services. Cultural stigma further constrains women with disabilities ability to pursue pregnancy and motherhood, as societal norms may undervalue their roles as wives and mothers [10]. Women with disabilities are also less likely to receive family planning counseling, antenatal care, and skilled birth attendance, which may increase their risk of preventable complications and mortality [8]. Given that other marginalized populations such as adolescent mothers, widows, and women with little education or income, often face compounded disadvantages in accessing respectful, high-quality MNH care [11], women with disabilities may experience unique barriers that shape access to care in distinct ways and merit increased scholarly attention. The World Health Organization’s Quality of Care Framework underscores that improving MNH outcomes requires health systems to address both access and experience of care, including the intersecting vulnerabilities that shape who is reached and how services are delivered [12]. Furthermore, international frameworks increasingly emphasize the need for disability-inclusive health systems. The United Nations Convention on the Rights of Persons with Disabilities (UNCRPD) affirms the right to the highest attainable standard of health without discrimination and acknowledges that women and girls with disabilities may face multiple and intersecting forms of discrimination [13]. Recent World Health Organization (WHO) guidance on health equity for people with disabilities further calls for integrating disability inclusion into health-sector planning, service delivery, and monitoring [14].

Global awareness of the challenges for women with disabilities is growing, but little is known about their experiences in Somalia. Existing evidence remains limited, and much of the available literature is regional rather than Somalia-specific. National sexual, reproductive, and maternal health policies have recognized disability inclusion to varying extents, however, disability is not sufficiently integrated into policy implementation or health information systems [15]. Furthermore, reliable data on the experiences of Somali women with disabilities during pregnancy, childbirth, and postpartum care remain limited [16]. The low availability of disability-disaggregated evidence may diminish the visibility of women with disabilities in MNH planning, monitoring, and quality improvement initiatives. Although tools such as the Washington Group Questionnaire [17] exist to facilitate disability measurement, there is limited disability-disaggregated evidence on MNH in Somalia. This gap may hinder the inclusion of women with disabilities in planning, monitoring, and quality improvement initiatives. This study addresses the evidence gap by examining the experiences of and perspectives on equitable access to MNH care among women with disabilities in Mogadishu, Somalia. By generating context-specific evidence on both access and experiences of care, the study will inform the development of inclusive service models and disability-responsive health policies aimed at reducing preventable maternal and newborn morbidity and mortality in Somalia and advancing equity objectives.

## Methods

### Study setting

The study was conducted in Mogadishu, the capital and largest city of Somalia, which has a high density of public and private health facilities and population estimates reaching close to 3 million, with about a third being internally displaced people [15]. Somalia’s health system is organized as a tiered service-delivery structure, in which maternal and newborn health services are distributed across primary and referral levels according to the complexity of care required. Routine antenatal, postnatal, and certain delivery services are typically offered at primary-level facilities, while cases involving complications are referred to higher-level facilities. Data collection took place in three public government health centers supported by the International Rescue Committee (IRC) in Mogadishu. Facilities were selected using convenience sampling, based on their inclusion in IRC programmes and the feasibility of conducting on-site interviews. The study sites were health centers that provided maternal and newborn health services within the wider health system in Mogadishu. The three centers differed in the extent to which they had structures relevant to women with disabilities (such as ramps, wide doorways, or adjustable beds), which allowed us to capture experiences across varying levels of physical accessibility.

### Study design

**Fig. 1:**
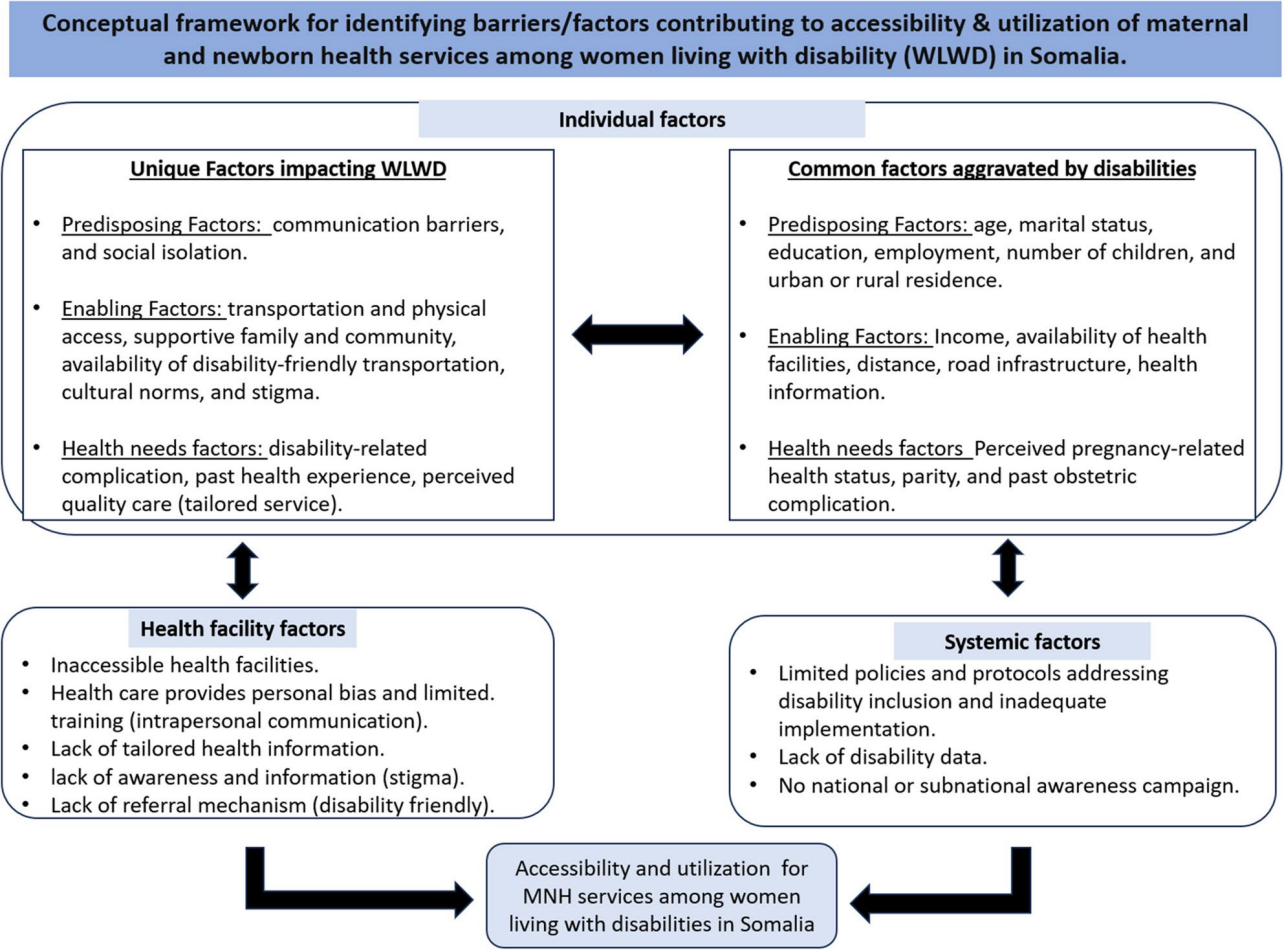
Conceptual framework adapted from Andersen’s Behavioral Model of Health Service Use [19] illustrating individual, facility, and systemic factors influencing access to and use of MNH services among women living with disability

A qualitative descriptive study design was used to examine respondents’ experiences accessing MNH care. This approach allowed participants to describe their experiences in their own voices and to capture aspects of access and quality of care that structured questionnaires might miss [18]. The study was informed by Andersen’s Behavioral Model of Health Service Use, which conceptualizes access to care as shaped by predisposing, enabling, and need-related factors (see Fig. 1) [19]. This framework guided the development of the interview topic guides and the overall approach to data collection and analysis. Data collection included semi-structured interviews with women with disabilities and their caregivers, as well as key informant interviews with representatives from disability and community-based organizations. This combination of client- and system-level perspectives allowed exploration of both individual experiences and institutional views on access to care.

### Sampling and recruitment

A purposive sampling strategy was used to recruit participants in both groups. This approach enabled the inclusion of information-rich participants with direct experience relevant to the research questions, including women with different disability experiences, caregivers, and stakeholders engaged in disability inclusion and maternal and newborn health services. Data collection continued until the research team determined that thematic sufficiency had been achieved, with no substantively new issues emerging within the main analytic domains during successive interviews. Client-level participants consisted of eight women aged 18–49 years with physical or visual impairments who had given birth within the past three years, and four caregivers of women with hearing impairments or intellectual disabilities who provided support during pregnancy, childbirth, and the postpartum period. Stakeholder-level participants included representatives from registered disability and community-based organizations that provided services or advocacy related to MNH care for women with disabilities (*n* = 5). These key informants were selected to provide insight into organizational practices, service delivery, and broader system-level barriers and facilitators. The sample size aligns with qualitative research guidance, which suggests that focused studies involving information-rich participants can generate sufficient depth to address specific research objectives [20]. Client-level participants were identified by trained midwives and community health workers (CHWs) working in IRC-supported health facilities and catchment communities. Before recruitment, midwives and CHWs completed a training session on study procedures, informed consent and disability-sensitive communication. They informed women with self-reported physical or visual impairments whom they had previously engaged through antenatal, intrapartum, or postpartum care, or community outreach about the study during routine facility or home visits. For women with hearing impairments or intellectual disabilities, midwives and CHWs instead approached a primary caregiver, identified by the household or the CHW as the main person involved in the women with disabilities care, to discuss the study and assess interest in participation. With the woman’s or caregiver’s permission, the research team was then provided with contact details to explain the study, confirm eligibility using an adapted version of the Washington Group Short Set of Questions [17] on functional limitations, and obtain informed consent.

Recruitment of stakeholder-level participants was undertaken in collaboration with the Federal Ministry of Health (FMoH). The research team compiled a list of registered disability and community-based organizations engaged in disability inclusion or reproductive, maternal, and newborn health, which the FMoH subsequently validated to confirm eligibility. From this list, five organizations were purposively selected based on their active MNH or advocacy program involving women with disabilities. Within each organization, the staff members responsible for disability inclusion or maternal health activities were invited and consented for an interview to provide perspectives on service delivery, system-level barriers and facilitators, and coordination with government and other partners. 

### Data collection

All interviews were conducted by the first author (MJ), a Somali-speaking qualitative researcher. Data was collected between July and September 2024 in private, accessible spaces within IRC-supported health facilities in Mogadishu to ensure comfort and confidentiality. Interviews were conducted privately with only the interviewer and participant present, unless the participant requested the presence of a caregiver or support person. In such cases, the accompanying individual was present solely at the participant’s request and exclusively to provide necessary support during the interview. Data were not collected directly from women with hearing impairments or intellectual disabilities because of the absence of a standardized Somali sign language and concerns about obtaining informed consent. Instead, their caregivers were interviewed to capture their experiences and observations while supporting women with disabilities, including access to services, communication with providers, and support needs during pregnancy, childbirth, and the postpartum period.

A semi-structured interview guide was developed from literature on disability-inclusive MNH, informed by Andersen’s Behavioral Model, and piloted before data collection. The guide for women with disabilities covered five domains: (1) demographic and disability screening using adapted Washington Group Questions [17] (2) pregnancy and birth experiences (3), access to and quality of MNH services (4), barriers and enablers of care, and (5) recommendations for improvement. Caregiver interviews followed a similar structure but focused on caregivers’ experiences and observations while supporting the woman with disability during her pregnancy, childbirth, and the postpartum period. Key informant interviews focused on organizational experience and perspectives related to disability-inclusive MNH care, including service delivery practices, system-level barriers and facilitators, disability inclusion in planning and implementation, and recommendations for improvement.

Each interview lasted approximately 45–60 min, was conducted in Somali, and was audio-recorded with participant consent. Field notes were used to document non-verbal expressions and contextual observations. For participants unable to provide written consent due to literacy or disability-related barriers, oral consent was obtained using an illustrated consent booklet with simplified Somali text and visual aids. Audio files were stored in encrypted, password-protected folders. Somali-language interviews were translated directly into English and transcribed by the first author (MJ). Transcripts were de-identified, assigned unique participant IDs, and reviewed against the original audio to ensure accuracy and preserve meaning. Somali terms with cultural significance were retained and italicized in English quotations, with brief explanations provided at first use.

### Data analysis

Deductive, iterative thematic analysis guided by an adapted version of Andersen’s Behavioral Model was used (see Fig. 1). Influences on MNH care were grouped into three analytic levels: individual, facility, and system. At the individual level, we examined women’s characteristics, resources, and perceived need for care. The facility level included service organization and provider-client interactions and the system level covered policies, governance, and disability-related data. The research team created a structured codebook using the adapted Andersen’s Behavioral Model and interview guides. Other operational codes, such as physical accessibility, provider attitudes, communication, referral processes, and disability-related policies, were identified and defined through group discussion before the coding process began.

Transcripts were coded in Dedoose (v10.0.35) [21] using a primarily deductive approach based on the agreed codebook and the analytic structure derived from Andersen’s Behavioral Model. Each transcript was reviewed at least once more to ensure coding consistency and identify any missed codes. When participants raised issues not adequately captured by the initial framework, additional codes were incorporated to ensure that relevant themes were not overlooked. Coded segments were grouped into sub-themes within each analytic level to identify patterns and contrasts. Themes were compared across women with disabilities, caregivers, and organizational representatives. All transcripts were coded using the same analytical approach; however, interviews with women with disabilities and caregivers contributed most directly to the development of individual- and facility-level themes, while key informant interviews contributed primarily to the development and interpretation of system-level themes.

After identifying gender-related themes in the initial analysis, we conducted a second analytical step using Morgan’s Gender Analysis Matrix, which was adapted from the gender-power relations domains outlined by Morgan et al. [22]. This method enabled us to examine how gender and disability influenced women’s experiences across four domains, with some domains relabeled to better align with the content of our interviews: access to resources, practices and participation, beliefs and perceptions, and decision-making and power. As the Matrix was introduced at a later stage of analysis, we did not apply the framework’s full component on the negotiation and transformation of power, as these aspects were not consistently addressed in the interviews. We re-coded and analyzed relevant data segments using the Gender Analysis Matrix to deepen our understanding of gender and structural influences. Final themes and sub-themes were refined through collaborative review sessions and team discussions to ensure coherence and credibility.

### Ethical considerations

This study received ethical approval from the International Rescue Committee Institutional Review Board, protocol H 1.00.065, and from the Somalia National Institute of Health and Ministry of Health, approval NIH/IRB/15/JUNE/2024. All procedures were conducted in accordance with the Declaration of Helsinki. Informed consent was obtained from all participants before data collection, and confidentiality was maintained throughout the study.

### Researcher reflexivity

The first author is a non-disabled Somali woman with training in medicine and public health. She shared language, culture, and social experiences with participants and possessed an understanding of Somali norms regarding marriage, motherhood, family decisions, and the meaning of disability. This background, along with her familiarity with local expressions and religious values, facilitated trust and enabled participants to discuss sensitive topics more openly. As an insider, the first author provided insight into the ways gender, disability, and health systems influenced women’s experiences with MNH care. Participants were recruited through IRC-supported facilities, and all authors, including the first author who collected the data, were affiliated with IRC. This facilitated access to women with disabilities who may otherwise have been difficult to reach, but it may also have influenced participant responses and introduced the possibility of researcher or respondent bias. At the same time, the authors had no role in service provision or supervision at the health facilities included in the study, and these facilities were not places of work for the authors. This meant there were no prior interactions between respondents and the authors, which may have helped reduce concerns about discussing negative experiences with health providers. The first author remained cognizant that her dual position as both insider and partial outsider could influence data collection and interpretation.

The broader research team included specialists in MNH, disability, and health systems research, whose varied perspectives strengthened the analytical process and encouraged critical reflection on emerging findings. Reflexive approaches, such as keeping field briefs, holding routine debriefings, and seeking peer input on early analyses, helped enhance the credibility and depth of the interpretation [23]. These measures facilitated the identification and mitigation of bias, thereby enhancing the credibility and transparency of the research.

## Results

The sociodemographic characteristics of women with disabilities are first described. Findings are subsequently organized according to the levels of Andersen’s Behavioral Model. Within each level, Morgan’s Gender Analysis Matrix is applied as a cross-cutting lens to examine how gender and disability influence these experiences. Findings are therefore presented in an integrated manner.

### Sociodemographic profile

This section summarizes the characteristics of women with disabilities represented in the study, including those interviewed directly and those described by caregivers (see Table 1). Most were aged 25–34 years, had low levels of formal education, and were unemployed. Many were divorced and had experienced multiple marriages, often within polygamous households. Early pregnancy was common, with half reporting a first pregnancy before 18 years of age. Most were financially dependent on relatives, with husbands most reported as heads of household. In addition, five key informants were interviewed, comprising representatives from disability organizations, community-based organizations, and government stakeholders engaged in disability inclusion and maternal and newborn health. Due to the limited number of such stakeholders in Mogadishu, we reported only general descriptors for this group to reduce the risk of indirect identification.

**Table 1:** Characteristics of women participants and caregiver-reported women (*n* = 12)

Variable	Category	*N* (%)
Age group	25–34 years	7 (58)
	35–44 years	5 (42)
Education	Quranic school	6 (50)
	No schooling	4 (33)
	Primary education	2 (17)
Marital status	Divorced	7 (58)
	Married	5 (42)
Marriage history	Married once	2 (17)
	Married 2–3 times	7 (58)
	Married 4 or more times	3 (25)
Rank in polygamous household	First wife	3 (25)
	Second wife	4 (33)
	Third or fourth wife	5 (42)
Head of household	Husband	8 (67)
	Brother	2 (17)
	Mother	2 (17)
Age at first pregnancy	< 18 years	6 (50)
	18–20 years	4 (33)
	≥ 21 years	2 (17)
Parity (number of pregnancies)	1–3	4 (33)
	4–6	3 (25)
	7 or more	5 (42)
Number of living children	1–2	2 (17)
	3–4	4 (33)
	5 or more	6 (50)
Occupation	Not employed	12 (100)

### Adapted andersen’s behavioral model

Thematic analysis identified several interconnected themes organized into three levels of Andersen’s Behavioral Model: individual, health facility, and system factors (see Table 2). This framework demonstrates that barriers and enablers function across multiple layers, ranging from women’s personal autonomy and household dynamics to broader structural and policy contexts.

**Table 2:** Themes organized by Andersen’s Behavioral Model domains

Domains	Themes
Individual factors	• Autonomy and interpersonal relationships (unpacked using Gender Analysis Matrix) • Marriage and Childbearing (unpacked using Gender Analysis Matrix) • Economic constraints (unpacked using Gender Analysis Matrix) • Poor transportation and referral system • Community attitudes
Health facility factors	• Inaccessible health infrastructure • Communication barriers • Stigma and provider attitudes
Systemic factors	• Limited inclusion in planning • Intersectoral barriers • Limited policies and resource allocation • Lack of disability data • Limited public awareness campaigns

### Individual factors

#### Autonomy and interpersonal relationships

Many respondents described limited autonomy and decision-making power in relation to their health, relationships, and care-seeking. Many participants indicated they had little influence over decisions related to their health, relationships, and care-seeking as family members or caregivers often made choices on their behalf. As one woman with a visual impairment stated, *“My family decides everything for me. Where I go*,* who to marry*,* and even if I can go to the facility to give birth.”* Although relatives frequently characterized this dependence as care or protection, women perceived it as a persistent limitation on their independence. Furthermore, disability influenced the ways in which women established relationships and developed trust within care environments. Several participants reported feeling safer at home, where family support was accessible, and expressed concerns about potential mistreatment by community members or health providers. Family members, neighbors, and close friends were crucial sources of practical and emotional support, especially when women needed help with mobility, labor and delivery support, and childcare. Women described these relationships as enabling them to cope with pregnancy and childbirth even when their independence was constrained. One participant recalled, *“During my delivery*,* my close friend supported the Traditional Birth Attendant (TBA) by holding both my legs tightly and telling me to relax so [the TBA] could do her job… she also gave me tea and fed my children.” (Woman with physical impairment)*.

*“Being blind*,* I feel more comfortable and supported at home*,* where my family can assist and understand me and treat me with respect without fearing they [health providers] will take advantage of my blindness and do something to me or my baby I don’t agree with.” (Woman with visual impairment).*

Participants reported reliance on family members and caregivers for decisions regarding marriage, mobility, and care-seeking. Husbands, mothers, and in-laws frequently served as primary decision-makers regarding marriage, mobility, and access to facility-based care. Limited educational opportunities further restricted women’s access to information and heightened their dependence on relatives when navigating services. Collectively, these findings indicate that women’s autonomy was frequently limited by dependence on others within household and care-seeking contexts.

#### Marriage and childbearing

Marriage and childbearing were described as central to women’s lives. Most respondents were divorced and had experienced multiple pregnancies. Many described multiple marriages as reflecting limited choice, with families arranging marriages, often to men who already had wives within extended family networks, to secure economic and caregiving support. Participants also described situations in which others made decisions about who they should marry and when they should have children. Many at the same time expressed the joy and recognition that came with motherhood. They noted how these experiences provided them with high self-esteem, confidence, a sense of normality, and completeness as women.

*“Having many children is seen by Somali women as a sign of family affluence and wealth…. I felt so happy when I got pregnant and eventually gave birth safely. My family and people now appreciate me*,* which makes me feel accomplished and fulfilled.”* (*Woman with physical impairment)*.

*“Being pregnant and able to give birth is a way to express that I am also a normal woman. Living with a disability*,* many people think that I am a naafo [a Somali term often used to describe a disability in a pitying*,* stigmatizing way*,* implying someone is incapable] who does not have feelings or a desire to be married*,* get pregnant*,* or raise a child. Instead*,* they want me to focus on my condition. So*,* if I can give birth and raise my child*,* it will allow me to prove wrong to all those who believe I am not a normal person simply because of my disability.” (Woman with physical impairment)*.

Others spoke of earning respect from families through childbearing. *“You have no idea how happy I felt when I gave birth; I was overjoyed. I wanted to have children to help continue the perpetuity of my husband’s clan*,* earn respect from his family and community members*,* and avoid being perceived as a burden.” (Woman with visual impairment)*.

Women with disabilities further described experiencing pressure to marry early and to have multiple children.

Both women and their relatives emphasized a belief that children would constitute an important source of future support, including emotional assistance and help with daily living activities.

One woman living with blindness said, *“My daughter is my eyes now. When I walk*,* she holds my hand and tells me what lies ahead. She’s only eight*,* but she’s my strength and sight.”*

Similarly, a family member explained, *“I want my daughter to give birth to many children so they can take care of her when I’m not around. Her 9-year-old girl is already supporting her in everything.” (Caregiver of woman with hearing impairment).* These narratives further demonstrate that daughters often assume caregiving responsibilities from an early age.

#### Economic constraints

Financial hardship was a recurring theme across all respondents, shaping whether and how women could access MNH services. Economic hardship limited access to resources, influenced care-seeking behaviors, and increased dependence on others.

All women were unemployed and dependent on family or community support; therefore, most of them struggled to cover basic costs associated with facility-based care, including transport to clinics, laboratory tests, medications, and even essential household utilities. One participant explained, *“I don’t work*,* and my family is poor. My husband provides for us financially*,* while my mother-in-law and I manage the home and care for the children. We don’t have enough money to spend on transportation and health services.” (Woman with physical impairment)*.

Many women relied on support and donations from family members, neighbors, and charitable organizations, which provided occasional cash or food assistance. A caregiver of a woman with a hearing impairment shared, *“My daughter is unemployed with three children from two different marriages. She cannot afford electricity or water… Our neighbors are helping us raise the children by sending money or food.”*

Economic insecurity also shaped the type of care women sought during childbirth. For some, home deliveries with TBAs were the only feasible option because they were cheaper, flexible with payments, and embedded in the community. As one woman with a physical impairment described, *“Home delivery costs little compared to the facility where we need to pay for transportation*,* laboratory*,* and other costs. The TBA takes whatever I offer her*,* maybe 3 to 5 dollars or some food.”* These accounts indicate that financial barriers restricted access to facility-based care and contributed to increased reliance on informal support and community-based providers.

#### Poor transportation and referral system

Transport was another major barrier. Women reported stories of how the inaccessibility and unaffordability of public transport, absence of disability-sensitive referral systems, and poor road infrastructure shaped their ability to access quality health services. For many, reaching a nearby facility requires depending on others for assistance, particularly among those with physical or visual impairments. One participant explained, *“I want to check on my pregnancy*,* but I’m struggling with how to get from my home to the health center. I can’t use my wheelchair to reach the center because there isn’t one nearby. The uneven pathways*,* their poor condition*,* and the lack of an ambulance for my situation are significant problems. These challenges have made me choose to deliver at home.” (Women with physical impairment)*.

Such barriers often pushed women to seek alternatives, such as home delivery or support from neighbors and family members. A woman with a physical impairment recounted being referred from the health center to a large hospital without any transport support or guidance. *“When I was told to go to another hospital*,* no one explained how to get there or what to do when I arrived. I was in pain*,* scared*,* and alone. I didn’t know if someone would help me when I got there. I thought*,* maybe it’s better to stay home and take the risk than to go through that again.”*

Transport challenges were not only logistical but also social. Several women described the embarrassment of using crowded public minibuses while visibly pregnant and disabled. A woman with a visual impairment shared, *“I always feel embarrassed when people look at me while I use public transportation*,* especially when I am pregnant. I often need help from someone or several people to get onto the minibus*,* which takes longer than I’d like. Also*,* our religion doesn’t permit strangers to touch women’s bodies*,* which makes me feel more embarrassed and I prefer to stay home.”* While women with disabilities often view pregnancy positively, dominant social and religious norms concerning bodily contact can make receiving public assistance during pregnancy especially uncomfortable.

Despite the barriers they faced, women also described a few enabling factors that supported their access to care. Living close to free or low-cost health facilities was seen as a major advantage, reducing both financial strain and the discomfort of public scrutiny. One participant shared, *“During my last two pregnancies*,* I moved to a different district with a free Maternal and Child Health center nearby. It made it convenient to check on my pregnancy and prevented me from running into people who always had something to say.” (women with physical impairment)*.

#### Community attitudes

Stigma and negative attitudes from the community were common, particularly toward pregnancy and motherhood among women with disabilities. Women described facing negative comments, disapproving looks, and hurtful statements from family and community members during pregnancy. These experiences suggested that some participants felt they were viewed by others as unfit for marriage, pregnancy, or parenting because of their disability. This scrutiny was described as generating fear, shame, and reluctance to seek healthcare services. This also contrasted with participants’ own descriptions of pregnancy and childbirth as experiences that could bring recognition and a sense of normality, particularly within close family networks or after a safe birth. However, participants suggested that such validation was often conditional and did not prevent negative treatment in public or from some community members.

One woman with physical impairment explained, *“When people see me outside*,* pregnant and sitting in a wheelchair*,* they give me bad looks and ask questions like*,* ‘Why did you get married? Why did you get pregnant? Who is the man who married this disabled woman?’ Some even say that I deserve to live like this.”*

Stigma was also reported by caregivers, who often tried to shield their daughters from public exposure. As one mother shared, *“I always think twice before taking my daughter outside when she is pregnant. I’ve heard people use her as a bad example in their conversations*,* claiming that if she got pregnant*,* anything is possible. Because of this*,* I avoid attending their discussions.”*

For women with visual impairment, community disbelief in their capacity to parent was especially painful. One participant recalled, *“Some community members believe that being blind makes you incapable of caring for your child*,* which is hurtful. Their doubt and negative comments every time they see me pregnant have made me feel sad and isolated.” (Woman with visual impairment)*.

### Health facility factors

#### Inaccessible health infrastructure

Most participants identified the inaccessibility of health facilities as a primary barrier to seeking and receiving quality care. For women with physical and visual impairments, the lack of ramps, wide entrances, and wheelchairs rendered facilities nearly impossible to navigate. Several participants reported experiencing humiliation when carried into buildings or examined in hallways due to inaccessible delivery rooms and laboratories. Such experiences undermined their sense of dignity and independence, and discouraged future use of health facilities, even when aware of the risks associated with home delivery.

One participant stated, *“Some health facilities only have stairs at the main entrances*,* so I had to be carried inside. There was no way I could go alone*,* and in some departments*,* such as laboratories*,* the narrow spaces do not accommodate my wheelchair. As a result*,* I had to be examined or tested in the corridors.” (Women with physical impairment)*.

The absence of a disability-inclusive infrastructure further exacerbated these challenges. Many respondents emphasized that the lack of adjustable beds, accessible toilets, and sufficient space for assistive devices constituted a significant gap in the provision of safe and respectful care.

### Communication barriers

Women with visual, hearing, and intellectual disabilities identified communication barriers with healthcare providers as a primary challenge when seeking care. These challenges were particularly pronounced during antenatal visits and childbirth, where effective communication is critical for patient safety and informed decision-making.

Several participants reported that healthcare providers frequently communicated exclusively with family members rather than addressing the women directly, resulting in confusion, anxiety, and an inability to express their needs. A caregiver of a woman with a hearing impairment reported, *“They (midwives) talk*,* and I try to follow. I shake my head to show I am listening*,* but I often do not understand what they mean.”* Another caregiver recounted, *“The midwife doesn’t understand her hand or face signs and cannot communicate with her. At the same time*,* she denied me [her mother) from accompanying her during birth and entering the delivery room. My daughter’s delivery experience was very bad and painful; we treated her for a bad injury for several months after her delivery.”*

Exclusion was also apparent during health education sessions. Participants with visual impairments reported that educational materials were inaccessible in the absence of verbal explanations or audio recordings, and no alternative formats were provided. One participant stated, *“I received a paper*,* but I am blind. I asked if it was available in audio format*,* but they said it was not. I depend on others to read information to me; however*,* in the health facility*,* no one helped. I sat through a health education session that I couldn’t see or hear.” (Women with vision disability)*.

Participants also described the lack of accessible information as limiting their understanding of available care options, including family planning. One visually impaired participant reflected, *“If I had known about family planning services*,* I would not have experienced nine pregnancies*,* especially since I cannot care for myself without support.”*

### Stigma and provider attitudes

All women with disabilities reported experiencing stigma and negative attitudes from healthcare providers. Participants indicated that these attitudes reflect the belief that women with disabilities should not become mothers or are incapable of caring for children. Participants characterized these experiences as emotionally detrimental and reported that they negatively influenced the quality of care provided.

Participants recounted being judged, dismissed, or treated with pity during antenatal visits and childbirth. Some were questioned about their right to be pregnant, while others were ignored in waiting areas or spoken to in condescending tones. A woman with a physical impairment recalled, *“When I went to the health center*,* the nurse looked at me and said*,* ‘Why would someone like you want to have a baby?’ I felt so small. They didn’t see me as a mother*,* only as a disabled woman. I waited while others were helped first*,* and no one told me anything. I left feeling ashamed*,* as if I had done something wrong just for being pregnant.”*

Family members also observed the effect of such treatment; one caregiver reported *“They treated her with pity*,* not respect. The midwife kept shaking her head during the examination*,* as if she couldn’t believe my daughter was there. I could feel the judgment in her face and tone. I didn’t want my daughter to go back*,* even though I knew she needed the care.” (Caregiver of a woman with intellectual disability)*.

Unlike physical barriers, stigma is not always visible. Women and their caregivers described subtle cues such as tone of voice, body language, or lack of eye contact, as well as overt behaviors like being denied support or rushed through consultations. A local organization representative explained, *“Many of the women we support are afraid to speak up during antenatal visits. They feel judged or misunderstood*,* especially those with hearing impairments or intellectual disabilities.”* Another added, *“Some women told us they avoid going to clinics because they can’t explain their symptoms clearly*,* and they fear being mistreated.”*

These perceptions were echoed by disability advocates, who pointed to the persistence of discriminatory beliefs among health workers. *“Some health workers still believe that disabled women shouldn’t be having children. One of our members was told by a nurse*,* ‘Why are you even pregnant?’ That kind of attitude pushes them away from care.”*

### Systemic factors

Interviews with disability organizational and MoH stakeholders, supported what women and caregivers reported and highlighted perceived structural and institutional barriers to inclusion.

#### Limited inclusion in planning

Stakeholders expressed weak governance and limited inclusive planning processes as barriers to disability-inclusive MNH services.

Stakeholders described disability rights as receiving public recognition, but reported that implementation often remained limited at facility and community levels. Representatives from disability organizations consistently reported exclusion from the design, budgeting, and evaluation of health programs, describing engagement as tokenistic and planning as largely top–down. One representative indicated, *“We are rarely consulted when the government or partners design health programmes. They often assume they know what people with disabilities need. If they had asked us*,* we would have told them that even getting through the door of a clinic is a challenge for many of our members.”*

Another stakeholder highlighted how limited system support compounds access barriers, noting, *“There is no one at the clinic to support them. Women are expected to come with someone*,* but that’s not always possible. So many end up not going at all.” These accounts suggested that disability inclusion was often not treated as a central consideration in health system design.*

#### Intersectoral barriers

Representatives explained how structural exclusion extends beyond the health sector, with intersectoral barriers shaping women’s long-term opportunities for independence and access to care. Education was frequently cited as an area where gender, disability, and poverty intersected to constrain girls’ and women’s opportunities.

Several participants described how these intersecting disadvantages excluded them from formal education and restricted their prospects for learning and independence. Participants described being withdrawn from school due to household financial constraints and gender prioritization of boys’ education, limiting their prospects for learning and future autonomy. One participant recalled, *“I went to school until class 5*,* then I stopped because my mother was the only one who was working… my mom and uncles decided to only pay for my brothers to complete their education. I was only allowed to go to Quran school until I memorized the Quran.” (Woman living with physical impairment)*. Another caregiver explained, *“My daughter never went to school because people said schools for disabled children are very expensive. We had no choice.” (Caregiver of woman living with hearing impairment).*

### Limited policies and resource allocation

Respondents reported that commitments to disability inclusion had not yet translated into practical implementation or dedicated funding. Most described disability inclusion in health strategies as being stated in principle but not clearly supported through dedicated funding. One local disability organization representative acknowledged this, stating, *“Even when disability is mentioned in national plans*,* it’s not budgeted for. So*,* it stays as a good intention.”* A major barrier was institutional fragmentation across ministries, with responsibilities spread across departments and no shared accountability. As one respondent from a local organization stated, *“Each ministry is doing its own thing. No one is coordinating*,* and there’s no shared vision for disability inclusion.”* Respondents described this lack of coordination as leading to repeated small, disconnected efforts and limiting the development of a sustained national approach to inclusive service delivery. Respondents also described disability inclusion as receiving lower priority than other urgent concerns, such as security, nutrition, and communicable diseases. Hence, funding for disability-related interventions remains inconsistent and often depends on short-term projects. As one disability organization representative explained, *“There are many groups trying to help*,* but without government leadership and resources*,* everything stays small and disconnected.”*

#### Gaps in disability data and monitoring

A central theme in stakeholder interviews was the lack of disability-disaggregated data within health information systems. Respondents identified this as a significant barrier to evidence-based planning. Respondents emphasized that without reliable data on the number, location, and service experiences of people with disabilities, particularly women in rural areas, it was difficult for decision-makers to identify service gaps or allocate resources effectively. *“We don’t know how many disabled women are giving birth at home or how many are denied care. We are working in the dark.” (Representative from disability organization)*.

Several stakeholders highlighted the absence of disability-related questions in national surveys and routine facility registers. Even when data is collected, representatives from disability organizations noted that the information is rarely analyzed or used for action. Respondents described the lack of disability-disaggregated data as contributing to the continued invisibility of people with disabilities in planning and programming. Stakeholders described this as a *“cycle of silence*,*”* in which the lack of data leads to limited funding, and limited funding further impedes data generation.

#### Limited public awareness campaigns

Stakeholders emphasized that stigma and discrimination remained important barriers and described broader social change as necessary alongside MNH programming. Limited national public awareness campaigns were identified as a key gap in efforts to address stigma and discrimination. Respondents argued that misconceptions about disability remained common among both health providers and the public and described these as contributing to women’s shame, fear, and avoidance of care. Participants stressed that awareness efforts should be led by people living with disabilities to challenge stereotypes and promote inclusion. As one Ministry of Health official stated, *“If people see a woman with a disability on the radio or TV talking about her life*,* it changes perceptions. But we don’t have that yet.”*

Local organizations described their efforts to address this gap through community sessions but noted that these initiatives remain limited in both reach and sustainability without government support. *“We have conducted small awareness sessions activities in our communities; however*,* we need government support to scale them up.” (One representative from disability organization)*.

## Discussion

This study found that women with disabilities often viewed pregnancy and childbirth as meaningful and affirming experiences, even though these were frequently shaped by stigma, dependence, and social exclusion. Participants also described pressure to marry and bear children at an early age, often within polygamous marriages and under conditions of limited autonomy over reproductive decision-making. Similar findings have been described in studies from Ghana, Nepal and South Africa, where motherhood offered disabled women a sense of dignity and recognition, yet their choices remained shaped, and often restricted, by patriarchal norms and financial dependence [10, 24, 25]. These studies also suggest that addressing such barriers needs more than awareness-raising activities. It requires impactful changes in community attitudes and improvements in the resources that support women’s independence. For example, these studies explain some approaches that involve working directly with community groups to address harmful ideas and misunderstandings about disability and sexuality [10, 24, 25]. Others focus on involving husbands and older family members, who often influence decisions about marriage and seeking care. Strengthening financial and social support for women with disabilities can also help reduce their reliance on others and limit outside control over their reproductive choices. At the service level, ensuring maternity care is inclusive and respectful is identified as essential for recognizing women with disabilities as decision-makers with the right to informed reproductive choice.

It is important to interpret these findings through an intersectional lens. The experiences of women with disabilities are shaped not only by disability, but also by the intersections of gender, poverty, marital instability, age, and prevailing social norms. Several constraints identified in this study, including limited autonomy in decisions regarding marriage, mobility, and care-seeking, may reflect broader gendered norms affecting women in Somalia rather than disability alone. However, disability often appeared to intensify these constraints by increasing women’s reliance on family members for transportation, communication, financial support, and daily assistance. Young, divorced women with disabilities, for example, appeared to be more often isolated and without financial support. Women with hearing impairments or intellectual disabilities encountered communication exclusion in addition to gendered expectations and household power imbalances. This interpretation is consistent with intersectional literature showing that social categories such as gender, disability, class, age, and marital status interact in ways that intensify disadvantage [26, 27]. Within this study, these intersecting barriers led many women with disabilities to perceive the health system as inaccessible on multiple levels: physical, social, and emotional. The findings on daughters assuming caregiving roles highlight the gendered distribution of unpaid care within households [28]. Although participants identified children as a source of future support, these findings indicate that caregiving responsibilities may be assigned to girls at an early age, thereby reinforcing broader societal expectations regarding gender and care.

Physical inaccessibility and communication barriers are closely linked and continue to limit the feasibility and acceptability of MNH care for women with disabilities. Structural barriers such as steps, narrow doorways, and non-adapted equipment suggest that these services are often not designed inclusively for women with disabilities. This discourages care-seeking and reinforces exclusion, which is consistent with evidence from other low- and middle-income countries showing that inaccessible facilities and lack of accommodation systematically limit access for people who use wheelchairs or assistive devices [29]. Communication barriers also undermine autonomy and safety. Many women in our study relied on relatives to interpret, which affects privacy and informed consent, and health providers often did not address them directly. Similar communication and information gaps have been documented in Bangladesh and Ghana, where the absence of interpretation services and accessible formats leads to misunderstanding and emotional distress [30]. Further, not allowing for labor companions increases fear and confusion, and raises concerns about consent, safety, and emotional well-being, especially for women who cannot communicate independently. Policies that support continuous labor support (a companion providing ongoing support throughout labor) are associated with better MNH outcomes, including improved birth experiences, lower caesarean rates, and fewer low Apgar scores [31]. These exclusionary practices are also inconsistent with WHO standards, which call for effective communication, respect, dignity, and the option of a companion of choice during labor and childbirth [32]. Evidence from other settings shows that these barriers are modifiable. For instance, providing accessible equipment, such as height-adjustable examination tables and wheelchair-accessible weighing scales, can help ensure that routine antenatal examinations are not missed, thereby improving the quality of care [33]. Tools co-developed with parents with learning disabilities, such as the Together Toolkit and Maternity Passport, can also offer feasible and patient-centered approaches to strengthen communication, continuity, and support women’s autonomy during maternity care; however, evidence on their impact on long-term clinical outcomes remains limited [31, 34]. Overall, our findings indicate that inaccessible communication, rather than disability itself, is a more immediate barrier to safe and respectful MNH care. This barrier restricts women’s ability to comprehend information, provide informed consent, and engage in decision-making. Our results further underscore the importance of adopting a universal design approach that incorporates clear verbal explanations and information in multiple formats as a system-wide quality improvement. Such measures can benefit women with disabilities, women with low literacy, adolescents, and others who require diverse communication supports.

Negative provider attitudes, including dismissive comments, impatience, and pity, may reflect broader systemic gaps in disability-inclusive and respectful maternity care rather than isolated interpersonal incidents. These attitudes may implicitly frame pregnancy among women with disabilities as illegitimate, making healthcare facilities unwelcome and contributing to delayed care-seeking and a preference for home birth. Existence of stigma regarding disabled women’s sexuality and motherhood is not limited to Somalia [29, 35]. There is need for integrated, actionable interventions that combine disability-adapted values clarification and attitude transformation with practical skills development in communication, consent, privacy, respectful care, and reasonable accommodations. Ideally, these interventions should be co-developed and co-delivered with women with disabilities, and reinforced through supportive supervision, routine monitoring, and accessible feedback and accountability mechanisms. Facility-level practice changes that safeguard privacy and dignity, such as the use of privacy screens, standardized consent communication, and permitting a companion of choice, may further address modesty-related concerns that contribute to the avoidance of facility births in other contexts [36, 37].

Respondents from disability organizations described disability inclusion commitments as having limited practical implementation and funding. In light of Somalia’s disability policy roadmap and broader national health and RMNCAH strategies [38, 39], these accounts suggest that disability inclusion is recognized at policy level but not yet clearly operationalized in ways that address the needs of women with disabilities seeking maternal and newborn health care. Although this study did not involve a formal policy analysis, the findings point to possible gaps between stakeholder concerns and the apparent translation of policy commitments into budgeting, implementation, and service delivery. Respondents also emphasized the absence of disability-disaggregated data in health information systems, which they viewed as a major barrier to inclusive planning and budgeting. This concern is echoed in Somalia’s 2024 National Disability Report [5], which highlights the importance of improved disability data for informing policy and programming. Better disability-disaggregated data could also support intersectional equity analysis by showing how disability interacts with gender, poverty, displacement, and age to identify groups at greater risk of exclusion and inform more targeted responses [40]. Stakeholders also identified public awareness campaigns as a potentially valuable strategy to reduce stigma and promote inclusion. However, these suggestions represent participant-informed recommendations rather than empirically validated interventions within the scope of this study.

The integration of Andersen’s Behavioral Model with Morgan’s Gender Analysis Matrix strengthened our analysis by identifying both the locations of barriers to care and the influence of gendered power relations on women’s experiences. Employing both frameworks advanced the analysis beyond a descriptive account of barriers. Future research on disability-inclusive maternal and newborn health in fragile settings may benefit from combining multi-level health systems frameworks with approaches that emphasize gender and power dynamics.

### Limitations

While the study protocol was developed following consultations with several disability advocacy groups and experts, it was not developed in partnership with women with disabilities. The study may have been strengthened by involving women with disabilities as a co-researcher to help shape the research questions and tools, advise on accessible and appropriate data-collection approaches, and contribute to the interpretation of findings to better reflect women with disabilities priorities and lived experience. For instance, the perspectives of women with hearing impairments or intellectual disabilities were primarily represented through caregivers. Nevertheless, triangulation of perspectives from women, caregivers, and stakeholders contributed to a coherent understanding of structural exclusion. A further strength of the study was the use of complementary analytical frameworks, which supported interpretation of women’s experiences across individual, facility, and system levels while also drawing attention to gendered power relations. The study also addresses an important evidence gap by examining disability-inclusive maternal and newborn health in a fragile, conflict-affected setting where such evidence remains limited. Social desirability bias may have occurred, as participants were referred by community health workers and midwives, potentially influencing the openness of their critiques regarding services. In addition, because the authors were affiliated with IRC, both participant responses and interpretation may have been influenced by this relationship, despite reflexive efforts during data collection and analysis. Additionally, the absence of interviews with health providers restricted triangulation of perspectives on communication barriers and quality of care. Finally, the sample was limited to selected IRC-supported facilities in urban Mogadishu, which may offer more accessible and higher-quality services compared to other settings such as rural areas and camps for internally displaced people.

## Conclusion

This study examined the experiences of and perspectives on equitable access to maternal and newborn health care among women with disabilities in Mogadishu, Somalia. The findings highlight significant barriers to safe and dignified care, including constrained autonomy, inaccessible infrastructure, communication barriers, provider stigma, and gaps in disability inclusion within health planning and information systems. These findings underscore significant deficiencies in inclusion and equity within the maternal health system. They also suggest the need for disability-inclusive infrastructure, co-design programming, provider training, accessible communication approaches, and stronger disability-disaggregated data systems. Support for caregivers who assist with mobility, communication, and care-seeking is also important to promote respectful accompaniment while protecting women’s privacy and decision-making. As context-specific evidence from Mogadishu, these findings can inform the development of more inclusive maternal and newborn health programming, policy dialogue, and service improvement in Somalia and other fragile contexts. Further research is warranted in other regions of Somalia, with increased inclusion of women with hearing or intellectual disabilities, and with a stronger focus on the quality, acceptability, and outcomes of care.

## Data Availability

Qualitative data is available from the corresponding author upon reasonable request and upon signature of a data-sharing agreement.
